# The effect of changes to GOLD severity stage on long term morbidity and mortality in COPD

**DOI:** 10.1186/s12931-018-0960-3

**Published:** 2018-12-12

**Authors:** Robert W. V. Flynn, Thomas M. MacDonald, James D. Chalmers, Stuart Schembri

**Affiliations:** Division of Molecular and Clinical Medicine, University of Dundee, Ninewells Hospital & Medical School, Dundee, DD1 9SY UK

**Keywords:** Chronic obstructive pulmonary disease, Global initiative for chronic obstructive lung disease (GOLD), Mortality, Morbidity

## Abstract

**Background:**

The Global Initiative for Chronic Obstructive Lung Disease (GOLD) severity stage classifies Chronic Obstructive Pulmonary Disease (COPD) into groups based on symptoms, exacerbations and forced expiratory volume in one second (FEV_1_). This allows patients to change to less severe COPD stages, a novel aspect of assessment not previously evaluated. We aimed to investigate the association between temporal changes in GOLD severity stage and outcomes in COPD patients.

**Methods:**

This was a record-linkage study using patients registered with a Scottish regional COPD network 2000–2015. Annual spirometry & symptoms were recorded and linked to healthcare records to identify exacerbations, hospitalisations and mortality. Spirometry, modified Medical Research Council (mMRC) dyspnoea scale and acute exacerbations over the previous year were used to assign GOLD severity at each visit. A time-dependent Cox model was used to model time to death. Secondary outcomes were respiratory specific mortality and hospitalisations. Effect sizes are expressed as Hazard Ratios HR (95%CI).

**Results:**

Four thousand, eight hundred and eighty-five patients (mean age 67.3 years; 51.3% female) with 21,348 visits were included. During a median 6.6 years follow-up there were 1530 deaths. For the secondary outcomes there were 712 respiratory deaths and 1629 first hospitalisations. Across 16,463 visit-pairs, improvement in COPD severity was seen in 2308 (14%), no change in 11,010 (66.9%) and worsening in 3145 (19.1). Compared to patients staying in GOLD stage A, those worsening had a stepwise increased mortality and hospitalisations.

**Conclusions:**

Improving COPD severity classification was associated with reduced mortality and worsening COPD severity was associated with increased mortality and hospitalisations. Change in GOLD group has potential as monitoring tool and outcome measure in clinical trials.

**Electronic supplementary material:**

The online version of this article (10.1186/s12931-018-0960-3) contains supplementary material, which is available to authorized users.

## Background

**Fig. 1:**
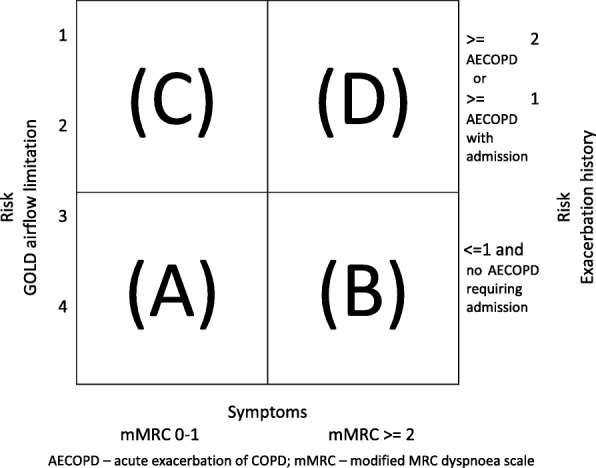
GOLD severity stage (2014) COPD classification system. AECOPD – acute exacerbation of COPD; mMRC – modified MRC dyspnoea scale

Unlike the 2007 Global initiative for Obstructive Lung Disease (GOLD) chronic obstructive pulmonary disease (COPD) severity stages that were based solely on Forced Expiratory Volume in one second (FEV_1_), the subsequent (2011 and beyond) GOLD COPD strategy statements classified COPD severity into distinct groups based on symptoms and exacerbations as well as spirometry (Fig. 1) [1, 2]. The classification of an individual patient’s COPD severity using the original GOLD strategy statements criteria would usually remain stable or indicate more severe disease because the natural progression of FEV_1_ often consists of a gradual decline over time (excluding any initial improvement after starting long acting bronchodilators or other treatment) [3]. The mean rate of decline in the ECLIPSE study was 33mls/year, although there were 8% of patients who increased their FEV_1_ by more than 20mls, it is unlikely that many of these would have improved sufficiently to be classified as having less severe disease using the previous stratification as this used broad percentage of predicted cut-offs [4]. However subsequent GOLD severity staging allowed patients to change to less severe COPD stages (e.g. D → A in the absence of exacerbations or improving symptoms) as well becoming more severe. The possibility of improved COPD severity over time was a novel aspect of COPD staging that had not been previously evaluated.

Already published data have shown that the new GOLD classification could improve the predictive value over FEV_1_ alone in relation to some poor outcomes and symptoms [5–7],; however, many of these studies have been cross-sectional in nature and several questions regarding severity shifts remain unanswered. Previous studies have looked into the stability of the new GOLD stages [8–10]; however, the relationship between change in GOLD stage and patients’ hospitalisations and mortality remained unclear especially as they move in between COPD severity stages several times over time.

The 2007 strategy statements were easily applied to clinical trial inclusion and exclusion criteria as FEV_1_ is relatively stable over a short time period. Improving the understanding of the changing nature of the new GOLD stages may aid the design of clinical studies aiming at individualised treatment beyond FEV_1_ alone.

It was traditionally thought that FEV_1_ trajectories did not change substantially with therapy [11] and therefore changes in GOLD 2007 stage were not an ideal end-point for clinical trials. With the inclusion of exacerbations and symptoms to the 2014 GOLD criteria, it is now possible to imagine that successful treatment would “downstage” COPD, by reducing exacerbation frequency and improving symptoms. We aimed to explore the validity of this concept in a longitudinal community-based COPD cohort.

This longitudinal observational study sought to establish whether changes in GOLD 2014 severity stage correlate with long term outcomes such as all-cause and respiratory-specific mortality, and respiratory specific hospital admission in COPD patients. Specifically, we sought to assess whether there was an association between change on GOLD stage and time to subsequent mortality, respiratory mortality and respiratory morbidity. It was designed and funded before the latest GOLD strategy statements whose severity classification separated spirometry from symptoms.

## Methods

This was a record-linkage cohort study using databases from Tayside Scotland. Data from the Tayside Medicines Monitoring Unit (MEMO) database is held within the Health Informatics Centre [12]. This system collects data from the Tayside region of Scotland; a compact geographical area with a population of over 400,000 people. Health care for the region is co-ordinated by Tayside Health Board, which maintains a computerised record of all patients registered with a general medical practitioner (GP). In brief, MEMO database contains several datasets including all dispensed community prescriptions, hospital discharge data, demographic data and biochemistry results. These data can be linked to disease-specific databases such as TARDIS database (Tayside Allergy and Respiratory Disease Information System) [13] and other routine clinical data, all of which are linked by a Community Health Index (CHI) number that is unique to each patient. All data was anonymised using Cleaning and Anonymisation by Mapping (CLAM) software and standard operating procedures (SOPs) as agreed by Tayside Committee on Research Medical ethics, Tayside Caldicott Guardians and research governance.

### Data sources

*CHI number master patient index* – this defined the study population from which subjects were identified, providing data on registered Tayside general practitioners, dates registered with general practitioners, date of birth and date of death.

*Scottish Morbidity Records 1 (SMR01)* – these data are routinely validated and collated by the Information and Services Division (ISD) of the National Health Service in Scotland and were available for Tayside from January 1, 1980 onwards [12]. These contained diagnostic and procedural codes relating to all hospital inpatient episodes of care using the International Classification of Diseases ninth or tenth revisions (ICD9, ICD10) [14, 15] and Office of Population Censuses and Surveys Classification of Interventions and Procedures (OCSP4) [16]. These data were used to identify respiratory specific hospital admissions during the study period as described below.

*TARDIS database* – The Tayside Allergy and Respiratory Disease Information System has been described before [13, 17]. Since 2001, general practitioners (GPs) in Tayside have been invited to refer patients in whom they suspect COPD for screening spirometry. This is carried out in the GP practices by COPD nurses after structured training in order to obtain standardized results. COPD was diagnosed in patients with a FEV_1_ < 80% of the predicted value (greatest of pre- and post-bronchodilator values) and FEV_1_/forced vital capacity (FVC) < 70% [17]. Those patients in whom COPD is diagnosed are then invited to participate in the structured management programme called TARDIS. Patients are invited annually and relevant measures are taken at the same time, including the post-bronchodilator FEV_1_ measure used in this study. If patients are housebound they are visited at home annually and this is also recorded in the database. A six-minute walk test is carried out in patients undergoing pulmonary rehabilitation.

### Study population

The study population was residents of Tayside, Scotland registered with a general practitioner between 1 January 2001 and 31 December 2015 (the latest date common to all the data source used). Subjects who left Tayside during the study period were censored at that point.

### Study subjects

Study subjects had spirometric confirmation of COPD (FEV_1_ / FVC < 70%) and were registered with the TARDIS database from January 2001 until the most recent mortality data available. They were required to be resident in Tayside for a minimum of 12-months before their qualifying COPD diagnosis. The date of the patient’s first annual spirometrically-confirmed COPD visit was used as the study entry date. Patients who had a cancer diagnosis prior to the diagnosis of COPD were excluded from the study (except for non-melanoma skin cancers). Patients who developed such cancers during the follow up time were censored 1 year prior to the diagnosis of cancer. A sensitivity analysis without this exclusion criteria relating to cancer history was performed. Patients with a single qualifying TARDIS visit only were excluded because more than one visit was required to assess change in spirometry and symptoms. Therefore, two visits with valid spirometry were required as a minimum for inclusion. This included patients who had one or more TARDIS visits, but with inadequate spirometry (FEV_1_ & FVC) for some visits.

### Outcomes

The primary outcome was all-cause mortality. Secondary outcomes were respiratory specific mortality and hospitalisations. Respiratory specific deaths were taken from the General Register Office (GRO) for Scotland death certification database where the primary underlying cause of death was a respiratory code (ICD10 code J00 – J99). Respiratory hospitalisation was obtained from SMR01, which contains all records of hospitalisations in Scotland. We included admissions coded using ICD10 codes that might be associated with an exacerbation of COPD hospital admission (J10-J18 “influenza & pneumonia”; J20-J22 “other acute lower respiratory infections”; J40–47 “Chronic lower respiratory disease”; J96 “Respiratory failure, not classified elsewhere”). For this endpoint patients were followed up until their first hospitalisation event. As a sensitivity analysis, we also considered all respiratory admission (ICD10 code J00 – J99) which is available in Additional file 1.

### Covariates

Covariates considered in the analysis consisted of other factors that could be potential confounders. These were: (1) Demographics (age, sex, social deprivation score, vaccination status, smoking history and Body Mass Index (BMI)); (2) Respiratory medication (inhaled corticosteroids, long acting beta-2 agonists, long acting antimuscarinics and other COPD specific medication (theophylline, aminophylline & carbocisteine); (3) Disease history: Cardiovascular Disease (CVD): primary prevention - hypertension (defined as on any anti-hypertension drug; treatment) and dyslipidaemia (defined as serum total cholesterol > 5 mmol/L). CVD history - myocardial infarction, heart failure, stroke, peripheral vascular disease (defined as hospitalization from the SMR1 database). Renal disease was defined as having serum creatinine ≥220 μmol/l [17]; (4) Charlson Comorbidity Index [18]; a measure of multiple co-morbidity derived from hospitalisation (SMR01) data and (5) Scottish Index of Multiple Deprivation (SMID), this is a measure of socioeconomic status that is assigned to study subjects at a postcode level.

### GOLD severity stage (2014)

This was established at the first qualifying visit and at all subsequent visits, using the patients’ risk of exacerbation and symptoms (Fig. 1).

Risk of exacerbation was based on two criteria:

1. Airflow limitation (from TARDIS spirometry – based on the current FEV1 expressed as a percentage of predicted FEV_1_.
2. Exacerbation history in the previous year. This involved two components:
  - The prescription of short courses of oral corticosteroids with or without antibiotics was used as a surrogate measure for exacerbation (derived from the prescribing data and considered an event if a dispensed prescription was for a course of oral prednisolone in which the total dose was in excess of 150 mg AND where the daily dose of prednisolone (where known) was equal to or greater than 30 mg). This definition was subject to a sensitivity analysis looking at the consistency of any findings where prednisolone was co-prescribed with pre-determined antibiotics (amoxicillin, co-amoxiclav, doxycycline and clarithromycin).
  - SMR01 hospitalisation, where the patient was admitted with a primary coded admission likely to be an acute COPD exacerbation (ICD10 J44 code) in the year prior to the visit. Again, this definition was subject to a sensitivity analysis to include a broader array of codes that might be associated with an exacerbation of COPD requiring hospital admission (J10-J18 “influenza & pneumonia”; J20-J22 “other acute lower respiratory infections”; J40–47 “Chronic lower respiratory disease”; J96 “Respiratory failure, not classified elsewhere”).

Symptoms were based on the modified MRC dyspnoea score (collected at each TARDIS visit). Based on all these criteria each visit had a GOLD severity stage assigned to it. The risk of exacerbation and symptoms was re-calculated approximately every 12 months (at each annual TARDIS visit) to calculate a “current” GOLD severity stage (2014). Where two visits occurred within a short space of time (within 3-months of each other) the visit showing the best spirometry data was used in the analysis. As a sensitivity analysis, this restriction was removed (i.e. with no minimum duration between visits specified) and broadened (to a minimum of 6-month between visits). Previous cohort studies have shown that GOLD stages B and C frequently have the least patients within them, and that the association with mortality in these groups overlaps. For these reasons, these groups were combined for the main analysis although a sensitivity analysis using all four GOLD 2014 stages was done.

### Change of GOLD severity stage

When considering how GOLD severity stage could change with time, a number of contingencies were possible. Tables 1 and 2 show the various classification systems that were trialled.

- Table 1 covers all possible change contingencies.
- GOLD stages B and C are the least common classification and their association with poor outcomes has overlapped in previous cohorts. An analysis with these 2 groups combined into one as shown in Table 2 was carried out.

**Table 1:** All possible GOLD severity stage (2014) changes between visits

A → A	B → A	C → A	D → A
A → B	B → B	C → B	D → B
A → C	B → C	C → C	D → C
A → D	B → D	C → D	D → D

**Table 2:** GOLD severity stage (2014) changes between visits (categories B & C combined)

A → A	BC → A	D → A
A → BC	BC → BC	D → BC
A → D	BC → D	D → D

### Statistical analysis

For initial analyses, a univariate non-time dependent analysis was used with patients followed up from the second of two visits until death, event or censored, with covariates taken at baseline only. This analysis used Kaplan Meier survival curves & log rank tests.

A Cox regression model using time-dependant analysis was used to explore the relationship between changing GOLD stage and the primary and secondary outcomes. This analysis took into account the fact that patients may change their GOLD severity during follow-up and allows the risk to be calculated using these continuously updated covariates. The time dependent variables (GOLD category) was updated at the time of each visit. Both univariate and multivariate analyses was carried out. The univariate analysis only takes into account the patients’ current and previous GOLD criteria. The multivariate analysis included all covariates considered to be potential confounders in an attempt to establish the true effect.

For both approaches, follow up started from the patients second qualifying visit as prior to this the patient had no baseline data against which to have changed from. For mortality outcomes, patients were followed up until death or censoring. For the respiratory hospitalisation outcome, patients were followed up until their first event only or censoring. Missing data were present for some covariates for a small number of patients (72 subjects with missing smoking history and 65 subjects with no socioeconomic status assigned). As these represented a small proportion of the cohort (less than 3% in total), it was assumed that these were completely missing at random and a complete case analysis performed for multivariate analyses.

To establish if there were any relevant interactions in the multivariate analyses, two-way interactions terms were modelled incorporating GOLD category change and other key covariates of interest, particularly age and sex. No such relevant interaction terms were found, and no interaction term were used in the final models.

Variables were included in the statistical models as potential predictors of the outcome events. All statistical analyses were carried out using SAS (version 9.4).

Studies of this nature frequently have to make assumptions regarding definitions of exclusions, exposures and outcomes. As detailed above, where such assumptions were made, these were the subject of sensitivity analyses which tested the reliability of any such arbitrary decisions that were made.

## Results

**Fig. 2:**
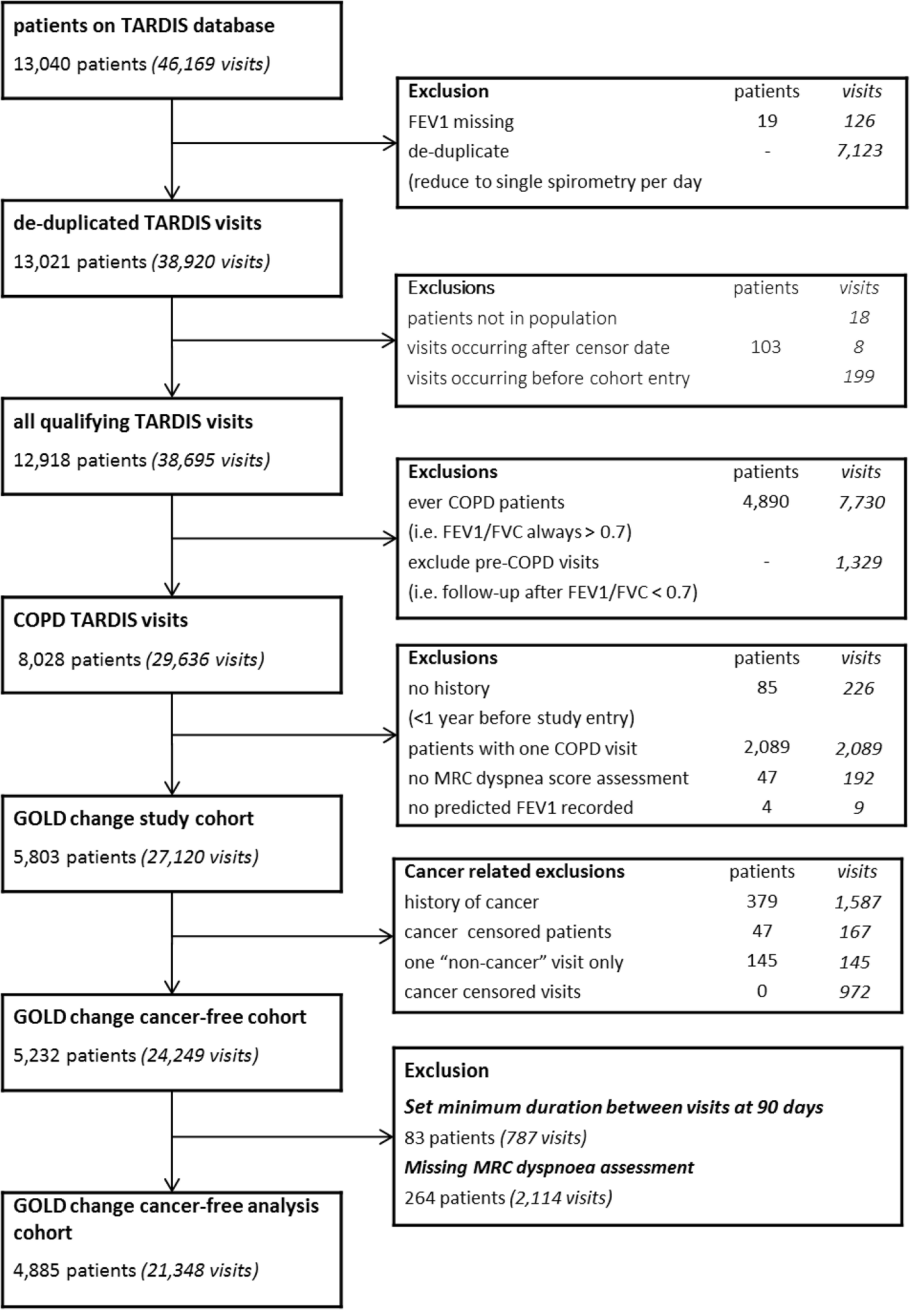
CONSORT diagram showing derivation of study cohort

Figure 2 shows the derivation of the study cohort. The main analysis cohort consisted of 4885 patients who had a total of 21,348 TARDIS visits during follow-up. The cohort was 51.4% female and had mean age of 67.3 years. There was a significant history of smoking amongst the study cohort with a median of 68.0 pack-years exposure. The cohort was disproportionately from more deprived backgrounds and the mean BMI was 26.3 (standardised deviation 5.3). A summary of the cohort characteristics is shown in Table 3.

**Table 3:** Baseline characteristics

	*n* = 4885
Age *(years)*	68 (60–75)
Female	2505 (51.3%)
BMI (kg/m2)^a^	25.7 (22.7–29.2)
Social deprivation^a^	
1 - most deprived	2091 (43.4%)
2	950 (19.7%)
3	547 (11.3%)
4	772 (16.0%)
5 - most affluent	460 (9.5%)
Rurality^a^	
Large Urban Areas	4357 (90.4%)
Other Urban Areas	164 (3.4%)
Small Towns	19 (0.4%)
Accessible Rural	280 (5.8%)
History of hypertension medication	2363 (48.4%)
History of dyslipidaemia	1645 (33.7%)
History of renal disease	99 (2.0%)
History of cardiovascular events	766 (15.7%)
History of cardiovascular prevention	3101 (63.5%)
Charlson Score	
0	3062 (62.7%)
1	1173 (24.0%)
2	393 (8.0%)
3	156 (3.2%)
4	70 (1.4%)
5	22 (0.5%)
over 6	9 (0.2%)
Smoking history *(pack years)*	38 (25–50)
Current smoking status^a^	
Current	1188 (24.3%)
Quitting^b^	1551 (31.8%)
Former	1830 (37.5%)
Never	310 (6.4%)
FEV_1_*(litres)*	1.54 (1.15–2.05)
FEV_1_ as percentage of predicted	68.1 (53.7–81.6)
Modified MRC dyspnoea score	1 (1–2)
Baseline GOLD stage	
A	2420 (49.5%)
B	1268 (26.0%)
C	476 (9.7%)
D	721 (14.8%)

*BMI* body mass index, *FEV*_1_ forced expiratory volume in one second, *MRC* Medical Research Council, *GOLD* Global Initiative for Chronic Obstructive Lung Disease

^a^Data missing for some patients: BMI for 139, social deprivation & rurality for 65, smoking pack years for 72, current smoking status for 6

^b^“quitting” means the patient is a current smoker who is either trying to or is contemplating giving up smoking

Data are median (IQR) or n (%)

There was a total of 31,891 years of follow-up to the primary endpoint (a median of 6.6 years per patient) and a median 1.1 years between consecutive TARDIS visits. There was a median of 4 visits per patient (minimum 2, maximum 12). The GOLD classification of each visit in the main cohort is shown in the Table 4.

**Table 4:** GOLD severity stage (2014) classification across all visits

GOLD category	First visit (i.e. 1 per patient)		All visits (up to 12 per patient)	
	*n* (visits)	%	*n* (visits)	%
A	2420	49.5	9741	45.6
B	1268	26.0	5344	25.0
C	476	9.7	2194	10.3
D	721	14.8	4069	19.0

**Fig. 3:**
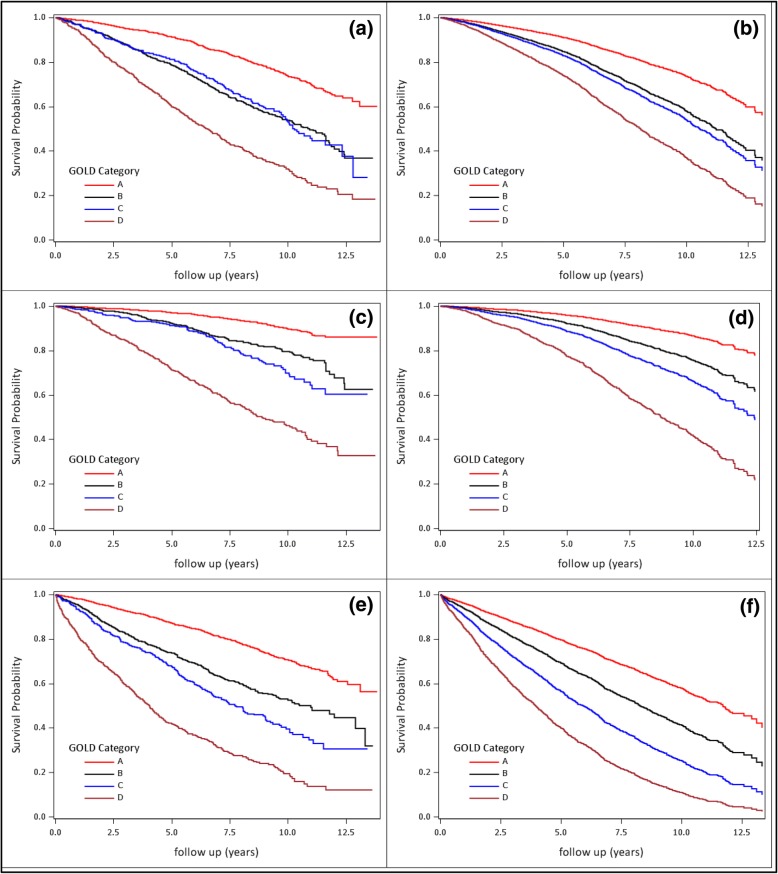
Univariate and multivariate (“adjusted”) Kaplan-Meier survival plots showing time to all-cause mortality from initial TARDIS visit. **a**, **c** & **e** show univariate plots, **b**, **d** & **f** are “adjusted” multivariate plots derived from a non-time dependent Cox model. The top two plots **a** & **b** are for the primary outcome (all-cause mortality); the middle two **c** & **d** for respiratory specific mortality; and the bottom two **e** & **f** for respiratory hospitalisation

In the primary analysis (all-cause mortality), there were 1530 primary outcome events. For the secondary outcomes there were 712 respiratory deaths and 1629 first hospitalisations. Figure 3 shows a description of the primary and secondary outcomes stratified by GOLD classification following both univariate and multivariate analyses. In general, it is seen that GOLD category A has the best survival, GOLD D has the worst outcomes, and GOLD B & C were in between with overlapping survival curves and similar hospitalisation outcomes. As GOLD C was a small group with similar outcomes to B, it was decided to pool these patients with the GOLD B patients for analysis.

GOLD severity changes were then elucidated. A summary of all the changes in GOLD category for the main analysis cohort is shown in Table 5. This shows the change in GOLD classified between the first two visits (with one change for each patient), and the change for all visits (with potentially multiple visits per patient).

**Table 5:** Summarised changes in GOLD category

GOLD category change	Change for first two visits only (i.e. 1 per patient)		Change between subsequent visits for all visits (up to 11 per patient)	
	*n* (visit pairs)	%	*n* (visit pairs)	%
A → A	1819	37.2	5802	35.2
A → B	369	7.6	1180	7.2
A → C	144	3.0	546	3.3
A → D	88	1.8	237	1.4
B → A	340	7.0	985	6.0
B → B	704	14.4	2375	14.4
B → C	44	0.9	139	0.8
B → D	180	3.7	609	3.7
C → A	119	2.4	404	2.5
C → B	34	0.7	115	0.7
C → C	221	4.5	765	4.7
C → D	102	2.1	434	2.7
D → A	50	1.0	130	0.8
D → B	124	2.5	406	2.5
D → C	86	1.8	268	1.6
D → D	461	9.4	2068	12.6
With GOLD categories B & C combined				
A → A	1819	37.2	5802	35.2
A → BC	513	10.5	1726	10.5
A → D	88	1.8	237	1.4
BC → A	459	9.4	1389	8.4
BC → BC	1003	20.5	3394	20.6
BC → D	282	5.8	1043	6.3
D → A	50	1.0	130	0.8
D → BC	210	4.3	674	4.1
D → D	461	9.4	2068	12.6

Table 6 shows the association between GOLD severity changes and the primary outcome (all-cause mortality) and secondary outcomes in a multivariate time-dependent analyses. In general, it shows that moving from a better to a worse GOLD category is associated with worse all-cause mortality, respiratory mortality and respiratory morbidity. There is also evidence of a “dose response” with larger changes in category being associated with a greater degree of risk.

**Table 6:** Survival analyses showing time to primary endpoints outcome (all-cause mortality) and secondary endpoints (respiratory specific mortality and respiratory hospitalisation) in a time-dependent multivariate analyses

GOLD Category Change	Primary outcome		Secondary outcome			
	Multivariate time dependent analyses		Respiratory specific mortality		Respiratory hospitalisation	
	HR (95% CI)	*P* value	HR (95% CI)	*P* value	HR (95% CI)	*P* value
A → A	1.00 ref		1.00 ref		1.00 ref	
A → BC	1.66 (1.34–2.07)	<.0001	2.28 (1.50–3.46)	0.0001	1.62 (1.31–2.01)	<.0001
A → D	2.08 (1.30–3.32)	0.0022	4.68 (2.37–9.23)	<.0001	3.13 (2.13–4.60)	<.0001
BC → A	1.58 (1.23–2.02)	0.0003	2.27 (1.43–3.60)	0.0005	1.44 (1.13–1.83)	0.0034
BC → BC	1.98 (1.67–2.35)	<.0001	3.27 (2.34–4.55)	<.0001	2.32 (1.97–2.73)	<.0001
BC → D	3.21 (2.61–3.94)	<.0001	7.51 (5.30–10.66)	<.0001	4.81 (3.98–5.82)	<.0001
D → A	1.31 (0.61–2.78)	0.4890	0.79 (0.11–5.70)	0.8112	1.01 (0.42–2.46)	0.9755
D → BC	2.96 (2.32–3.78)	<.0001	7.38 (4.99–10.93)	<.0001	4.12 (3.29–5.17)	<.0001
D → D	4.31 (3.64–5.10)	<.0001	12.86 (9.47–17.46)	<.0001	5.87 (4.97–6.92)	<.0001

In a model that also adjusted for age, sex, smoking history, BMI, use of COPD related medication at baseline, history of cardiovascular events, history of cardiovascular protecting therapies, renal failure, Charlson comorbidity index and socioeconomic status

The results from the various sensitivity analyses are given in the Additional file 1. These show the study findings to be robust to the various assumptions that where made when compiling the datasets and assigning GOLD stage categories.

## Discussion

A major aim of the change in GOLD classification to include symptoms and exacerbation history was the appreciation that spirometry alone did not capture the full complexity and heterogeneity of the COPD patient [19–21]. Although guiding pharmacotherapy was not the primary intention, this is now a core part of the guidance [2]. Guideline concordant therapy is difficult without understanding the propensity of patients to change severity stage. Understanding the stage natural progression and the associations with outcome of this is important to manage patients as without this understanding, changes to patients’ severity stage can make optimal management difficult.

In this manuscript, we describe the largest longitudinal cohort looking at variability of GOLD severity stage over time and for the first to describe the association of GOLD severity stage change with poor outcomes. As seen in other cohorts, GOLD severity stage C is the least common severity stage in our study. Our study confirms that GOLD A severity has the best survival, GOLD D has the worst outcomes [22], and that outcomes of patients with GOLD B and C severity lie in between and often overlap [5–7]. In keeping with previous studies, we show that over half patients’ severity stage remains stable [10]. Looking at those patients over time we show that GOLD stage A patients who remain in that stage have the best outcomes in terms of hospitalisations and mortality. Patients whose severity stage worsens have worse outcomes with a “dose effect” present such that worse outcomes are seen in those whose severity stage deteriorates by more than one step, for example A to D, when compared to those whose severity stage only worsens by one stage, for example A to B, or B to C, or A to BC). Patients who move from stage BC to A have worse outcomes than those who are only ever stage A, implying the latter have more stable disease and hence better outcomes. The converse is true at the other end of the severity scale. Interestingly, we have shown that improvement of COPD severity stage as described by the GOLD 2014 classification was associated with improved all-cause mortality, improved respiratory specific mortality and respiratory hospitalisations when compared to those individuals who remain in the same severity group. Our results may seem axiomatic; the only way to worsen one’s GOLD severity group is either by having more symptoms and/or more exacerbations and both factors have been repeatedly demonstrated to be associated with increased risk of mortality [13]. However, while the effects of individual risk factors have been previously studied, this is the first time such a change has been shown within a combined severity classification.

The strengths of this study are that data were collected in routine care with minimal exclusions, which increases the likelihood that the results will be applicable to other populations. This is significant as most patients with COPD are managed in primary rather than secondary care. The primary-care based nature of our cohort is demonstrated by the significant number of patients with GOLD stage A disease, unlike hospital generated cohorts which are less likely to be generalizable to the broader COPD population. Another strength is the significant representation of women which is not always the case in hospital-based cohorts.

Weaknesses of this study include the ability to only assess symptoms using mMRC dyspnoea score. This is in line with other reported cohorts [10]. Despite data being collected prospectively according to predefined criteria, follow-up data are observational and therefore prone to the weaknesses of this type of study. Another important consideration is that the latest GOLD classification (2017) separates the spirometry and exacerbation classifications, thus creating a total of 16 potential severity classifications [23]. This means that there are now 256 potential stage shifts (including stability). We attempted repeating our analyses with this classification however there were too many groups with very few or no individuals making models uninterpretable. Though we attempted to limit bias by adjusting for other potential confounders, bias due to unrecorded factors may remain.

## Conclusions

In summary, our data based on a large cohort of well-characterized community COPD patients provide important information on the assessment of patients with COPD. Using the new multidimensional GOLD classification, we showed that disease progression as defined by worsening GOLD stage is associated with poorer outcomes, whilst the reverse is true for improvement.

## Additional file

Additional file 1: Results tables for sensitivity analyses testing the assumptions made in the main analysis (as described in the methods). (DOCX 38 kb)

## Abbreviations

BMI: Body mass index
CHI: Community health index
CLAM: Cleaning and Anonymisation by Mapping
COPD: Chronic obstructive pulmonary disease
CVD: Cardiovascular disease
FEV1: Forced expiratory volume in one second
FVC: Forced vital capacity
GOLD: Global initiative for chronic obstructive lung disease
GP: General medical practitioner
HR: Hazard ratios
ICD10: International classification of diseases tenth revision
ICD9: International classification of diseases ninth revision
ISD: Information and Services Division
MEMO: Medicines Monitoring Unit
mMRC: modified Medical Research Council
OCSP4: Office of Population Censuses and Surveys Classification of Interventions & Procedures Fourth Revision
SMID: Scottish index of multiple deprivation
SMR01: Scottish morbidity records 1
SOPs: Standard operating procedures
TARDIS: Tayside allergy and respiratory disease information system
