# Supplementary material for: The effect of changes to GOLD severity stage on long term morbidity and mortality in COPD
Source: Respir Res. 2018 Dec 12;19:249. doi: 10.1186/s12931-018-0960-3 (PMC6291946; doi:10.1186/s12931-018-0960-3)
Supplement: Supplementary file 1 — Results tables for sensitivity analyses testing the assumptions made in the main analysis (as described in the methods). (DOCX 38 kb) [file 12931_2018_960_MOESM1_ESM.docx]

**Sensitivity analysis cohort: including cancer patients**

|  | **Primary outcome** | | **Secondary outcome** | | | |
| --- | --- | --- | --- | --- | --- | --- |
|  | All-cause mortality  n event = 2,431 | | Respiratory specific mortality  n event = 818 | | Respiratory hospitalisation  n event = 1,979 | |
| GOLD Category Change | HR (95% CI) | P value | HR (95% CI) | P value | HR (95% CI) | P value |
| A → A | 1.00 ref |  | 1.00 ref |  | 1.00 ref |  |
| A → BC | 1.42 (1.21-1.67) | <.0001 | 1.94 (1.34-2.82) | 0.0005 | 1.55 (1.28-1.87) | <.0001 |
| A → D | 1.86 (1.34-2.58) | 0.0002 | 3.57 (1.93-6.58) | <.0001 | 2.68 (1.90-3.79) | <.0001 |
| BC → A | 1.34 (1.12-1.61) | 0.0016 | 1.89 (1.25-2.86) | 0.0026 | 1.45 (1.18-1.79) | 0.0005 |
| BC → BC | 1.66 (1.47-1.89) | <.0001 | 3.06 (2.30-4.07) | <.0001 | 2.24 (1.94-2.58) | <.0001 |
| BC → D | 2.50 (2.14-2.93) | <.0001 | 6.00 (4.40-8.20) | <.0001 | 4.54 (3.83-5.38) | <.0001 |
| D → A | 1.57 (0.98-2.52) | 0.0606 | 1.64 (0.52-5.23) | 0.4014 | 1.13 (0.56-2.27) | 0.7384 |
| D → BC | 2.24 (1.85-2.71) | <.0001 | 6.13 (4.33-8.69) | <.0001 | 3.98 (3.26-4.86) | <.0001 |
| D → D | 3.09 (2.72-3.51) | <.0001 | 10.80 (8.29-14.05) | <.0001 | 5.22 (4.51-6.04) | <.0001 |

model also adjusts for: age, sex, smoking history, BMI, use of COPD related medication at baseline, history of cardiovascular events, history of cardiovascular protecting therapies, renal failure, Charlson comorbidity index and socioeconomic status

**Main analysis cohort: alternative definition of exacerbation including antibiotic prescribing**

|  | **Primary outcome** | | **Secondary outcome** | | | |
| --- | --- | --- | --- | --- | --- | --- |
|  | All-cause mortality  n event = 1,477 | | Respiratory specific mortality  n event = 686 | | Respiratory hospitalisation  n event = 1,578 | |
| GOLD Category Change | HR (95% CI) | P value | HR (95% CI) | P value | HR (95% CI) | P value |
| A → A | 1.00 ref |  | 1.00 ref |  | 1.00 ref |  |
| A → BC | 1.66 (1.34-2.05) | <.0001 | 2.36 (1.58-3.53) | <.0001 | 1.69 (1.38-2.07) | <.0001 |
| A → D | 2.08 (1.31-3.33) | 0.0021 | 4.75 (2.42-9.34) | <.0001 | 3.37 (2.30-4.95) | <.0001 |
| BC → A | 1.51 (1.18-1.92) | 0.0009 | 2.14 (1.36-3.38) | 0.0011 | 1.36 (1.07-1.74) | 0.0128 |
| BC → BC | 1.93 (1.63-2.28) | <.0001 | 3.33 (2.42-4.58) | <.0001 | 2.37 (2.02-2.77) | <.0001 |
| BC → D | 3.24 (2.65-3.98) | <.0001 | 7.53 (5.33-10.64) | <.0001 | 4.99 (4.12-6.04) | <.0001 |
| D → A | 1.04 (0.43-2.53) | 0.9263 | 0.90 (0.13-6.53) | 0.9189 | 1.72 (0.81-3.64) | 0.1590 |
| D → BC | 2.97 (2.32-3.81) | <.0001 | 6.96 (4.68-10.35) | <.0001 | 4.00 (3.17-5.05) | <.0001 |
| D → D | 4.25 (3.60-5.02) | <.0001 | 13.04 (9.68-17.57) | <.0001 | 5.81 (4.93-6.84) | <.0001 |

model also adjusts for: age, sex, smoking history, BMI, use of COPD related medication at baseline, history of cardiovascular events, history of cardiovascular protecting therapies, renal failure, Charlson comorbidity index and socioeconomic status

***Main analysis cohort: alternative definition of exacerbation including extended SMR01 codes***

|  | **Primary outcome** | | **Secondary outcome** | | | |
| --- | --- | --- | --- | --- | --- | --- |
|  | All-cause mortality  n event = 1,477 | | Respiratory specific mortality  n event = 686 | | Respiratory hospitalisation  n event = 1,578 | |
| GOLD Category Change | HR (95% CI) | P value | HR (95% CI) | P value | HR (95% CI) | P value |
| A → A | 1.00 ref |  | 1.00 ref |  | 1.00 ref |  |
| A → BC | 1.68 (1.35-2.10) | <.0001 | 2.36 (1.54-3.62) | <.0001 | 1.68 (1.36-2.08) | <.0001 |
| A → D | 2.46 (1.62-3.72) | <.0001 | 5.28 (2.80-9.95) | <.0001 | 3.32 (2.27-4.85) | <.0001 |
| BC → A | 1.54 (1.20-1.98) | 0.0008 | 2.21 (1.37-3.56) | 0.0012 | 1.50 (1.18-1.91) | 0.0009 |
| BC → BC | 2.01 (1.69-2.39) | <.0001 | 3.38 (2.40-4.74) | <.0001 | 2.31 (1.96-2.72) | <.0001 |
| BC → D | 3.19 (2.59-3.92) | <.0001 | 7.74 (5.42-11.06) | <.0001 | 4.97 (4.11-6.01) | <.0001 |
| D → A | 1.76 (0.93-3.33) | 0.0813 | 3.08 (1.11-8.58) | 0.0313 | 0.91 (0.38-2.22) | 0.8428 |
| D → BC | 2.97 (2.33-3.80) | <.0001 | 7.14 (4.78-10.67) | <.0001 | 4.29 (3.43-5.37) | <.0001 |
| D → D | 4.39 (3.70-5.20) | <.0001 | 13.33 (9.74-18.24) | <.0001 | 5.98 (5.07-7.06) | <.0001 |

model also adjusts for: age, sex, smoking history, BMI, use of COPD related medication at baseline, history of cardiovascular events, history of cardiovascular protecting therapies, renal failure, Charlson comorbidity index and socioeconomic status

**Main analysis cohort: alternative definition of exacerbation including antibiotic prescribing & extended SMR01 codes**

|  | **Primary outcome** | | **Secondary outcome** | | | |
| --- | --- | --- | --- | --- | --- | --- |
|  | All-cause mortality  n event = 1,477 | | Respiratory specific mortality  n event = 686 | | Respiratory hospitalisation  n event = 1,578 | |
| GOLD Category Change | HR (95% CI) | P value | HR (95% CI) | P value | HR (95% CI) | P value |
| A → A | 1.00 ref |  | 1.00 ref |  | 1.00 ref |  |
| A → BC | 1.66 (1.34-2.06) | <.0001 | 2.46 (1.63-3.70) | <.0001 | 1.75 (1.43-2.15) | <.0001 |
| A → D | 2.49 (1.66-3.74) | <.0001 | 5.17 (2.76-9.71) | <.0001 | 3.55 (2.43-5.18) | <.0001 |
| BC → A | 1.46 (1.14-1.88) | 0.0028 | 2.02 (1.25-3.25) | 0.004 | 1.41 (1.11-1.80) | 0.0052 |
| BC → BC | 1.96 (1.65-2.32) | <.0001 | 3.39 (2.44-4.70) | <.0001 | 2.35 (2.00-2.76) | <.0001 |
| BC → D | 3.20 (2.60-3.93) | <.0001 | 7.73 (5.45-10.97) | <.0001 | 5.13 (4.23-6.22) | <.0001 |
| D → A | 1.57 (0.80-3.06) | 0.1866 | 3.86 (1.54-9.71) | 0.0041 | 1.46 (0.69-3.09) | 0.3285 |
| D → BC | 2.96 (2.31-3.78) | <.0001 | 6.72 (4.49-10.07) | <.0001 | 4.23 (3.37-5.32) | <.0001 |
| D → D | 4.29 (3.63-5.08) | <.0001 | 13.40 (9.89-18.16) | <.0001 | 5.91 (5.02-6.97) | <.0001 |

model also adjusts for: age, sex, smoking history, BMI, use of COPD related medication at baseline, history of cardiovascular events, history of cardiovascular protecting therapies, renal failure, Charlson comorbidity index and socioeconomic status

**Sensitivity analysis cohort: no minimum gap between consecutive TARDIS visits**

|  | **Primary outcome** | | **Secondary outcome** | | | |
| --- | --- | --- | --- | --- | --- | --- |
|  | All-cause mortality  n event = 1,486 | | Respiratory specific mortality  n event = 691 | | Respiratory hospitalisation  n event = 1,596 | |
| GOLD Category Change | HR (95% CI) | P value | HR (95% CI) | P value | HR (95% CI) | P value |
| A → A | 1.00 ref |  | 1.00 ref |  | 1.00 ref |  |
| A → BC | 1.71 (1.38-2.12) | <.0001 | 2.24 (1.48-3.40) | 0.0001 | 1.62 (1.32-2.00) | <.0001 |
| A → D | 2.10 (1.32-3.36) | 0.0019 | 5.10 (2.66-9.79) | <.0001 | 3.32 (2.27-4.84) | <.0001 |
| BC → A | 1.56 (1.22-2.00) | 0.0004 | 2.06 (1.29-3.31) | 0.0026 | 1.42 (1.12-1.81) | 0.0045 |
| BC → BC | 2.00 (1.68-2.38) | <.0001 | 3.25 (2.34-4.52) | <.0001 | 2.30 (1.96-2.70) | <.0001 |
| BC → D | 3.24 (2.64-3.98) | <.0001 | 7.48 (5.28-10.59) | <.0001 | 4.76 (3.94-5.75) | <.0001 |
| D → A | 1.36 (0.64-2.89) | 0.4255 | 0.80 (0.11-5.83) | 0.8294 | 0.81 (0.30-2.18) | 0.6757 |
| D → BC | 3.05 (2.39-3.88) | <.0001 | 7.33 (4.97-10.81) | <.0001 | 4.16 (3.33-5.19) | <.0001 |
| D → D | 4.41 (3.72-5.22) | <.0001 | 12.79 (9.44-17.33) | <.0001 | 5.83 (4.95-6.86) | <.0001 |

model also adjusts for: age, sex, smoking history, BMI, use of COPD related medication at baseline, history of cardiovascular events, history of cardiovascular protecting therapies, renal failure, Charlson comorbidity index and socioeconomic status

***Sensitivity analysis cohort: 6-month minimum gap between consecutive TARDIS visits***

|  | **Primary outcome** | | **Secondary outcome** | | | |
| --- | --- | --- | --- | --- | --- | --- |
|  | All-cause mortality  n event = 1,470 | | Respiratory specific mortality  n event = 683 | | Respiratory hospitalisation  n event = 1,571 | |
| GOLD Category Change | HR (95% CI) | P value | HR (95% CI) | P value | HR (95% CI) | P value |
| A → A | 1.00 ref |  | 1.00 ref |  | 1.00 ref |  |
| A → BC | 1.64 (1.32-2.04) | <.0001 | 1.95 (1.28-2.97) | 0.0019 | 1.56 (1.27-1.93) | <.0001 |
| A → D | 2.06 (1.29-3.30) | 0.0025 | 4.30 (2.19-8.46) | <.0001 | 2.88 (1.94-4.29) | <.0001 |
| BC → A | 1.57 (1.23-2.02) | 0.0003 | 2.07 (1.30-3.28) | 0.0021 | 1.42 (1.11-1.81) | 0.0049 |
| BC → BC | 1.94 (1.63-2.31) | <.0001 | 3.00 (2.17-4.15) | <.0001 | 2.27 (1.93-2.66) | <.0001 |
| BC → D | 3.23 (2.64-3.96) | <.0001 | 7.18 (5.11-10.08) | <.0001 | 4.83 (4.00-5.82) | <.0001 |
| D → A | 1.30 (0.61-2.76) | 0.4977 | 0.73 (0.10-5.29) | 0.7552 | 0.81 (0.30-2.17) | 0.6728 |
| D → BC | 2.96 (2.32-3.79) | <.0001 | 6.93 (4.71-10.18) | <.0001 | 4.09 (3.26-5.12) | <.0001 |
| D → D | 4.23 (3.57-5.00) | <.0001 | 11.72 (8.70-15.78) | <.0001 | 5.71 (4.85-6.73) | <.0001 |

model also adjusts for: age, sex, smoking history, BMI, use of COPD related medication at baseline, history of cardiovascular events, history of cardiovascular protecting therapies, renal failure, Charlson comorbidity index and socioeconomic status

***Main analysis cohort: alternative definition of hospitalised outcome (extended SMR01 codes)***

|  | **Primary outcome** | | **Secondary outcome** | | | |
| --- | --- | --- | --- | --- | --- | --- |
|  | All-cause mortality  n event = | | Respiratory specific mortality  n event = | | Respiratory hospitalisation  n event = 1687 | |
| GOLD Category Change | HR (95% CI) | P value | HR (95% CI) | P value | HR (95% CI) | P value |
| A → A | - | - | - | - | 1.00 ref |  |
| A → BC | - | - | - | - | 1.49 (1.22-1.83) | 0.0001 |
| A → D | - | - | - | - | 2.91 (2.01-4.22) | <.0001 |
| BC → A | - | - | - | - | 1.34 (1.07-1.69) | 0.012 |
| BC → BC | - | - | - | - | 2.17 (1.86-2.53) | <.0001 |
| BC → D | - | - | - | - | 4.41 (3.68-5.28) | <.0001 |
| D → A | - | - | - | - | 0.86 (0.36-2.09) | 0.7442 |
| D → BC | - | - | - | - | 3.60 (2.89-4.48) | <.0001 |
| D → D | - | - | - | - | 5.22 (4.46-6.10) | <.0001 |

model also adjusts for: age, sex, smoking history, BMI, use of COPD related medication at baseline, history of cardiovascular events, history of cardiovascular protecting therapies, renal failure, Charlson comorbidity index and socioeconomic status
